# MicroRNAs as Important Players in Host–Adenovirus Interactions

**DOI:** 10.3389/fmicb.2017.01324

**Published:** 2017-07-17

**Authors:** Diogo Piedade, José M. Azevedo-Pereira

**Affiliations:** Host-Pathogen Interaction Unit, iMed.ULisboa, Faculdade de Farmácia, Universidade de Lisboa Lisboa, Portugal

**Keywords:** microRNA, adenoviruses, pathogenesis, gene regulation, mRNA translation, oncogenesis

## Abstract

MicroRNAs (miRNAs) are powerful regulators of gene expression and fine-tuning genes in all tissues. Cellular miRNAs can control 100s of biologic processes (e.g., morphogenesis of embryonic structures, differentiation of tissue-specific cells, and metabolic control in specific cell types) and have been involved in the regulation of nearly all cellular pathways. Inherently to their involvement in different physiologic processes, miRNAs deregulation has been associated with several diseases. Moreover, several viruses have been described as either, avoid and block cellular miRNAs or synthesize their own miRNA to facilitate infection and pathogenesis. Adenoviruses genome encodes two non-coding RNAs, known as viral-associated (VA) RNA_I_ and VA RNA_II_, which seem to play an important role either by blocking important proteins from miRNA pathway, such as Exportin-5 and Dicer, or by targeting relevant cellular factors. Drastic changes in cellular miRNA expression profile are also noticeable and several cellular functions are affected by these changes. This review focuses on the mechanisms underlying the biogenesis and molecular interactions of miRNAs providing basic concepts of their functions as well as in the interplay between miRNAs and human adenoviruses.

## Introduction

MicroRNAs (miRNAs) are small non-coding RNAs with approximately 22-nucleoides that regulate gene expression (Doench, 2003; Bartel, 2004; Kloosterman and Plasterk, 2006; Bushati and Cohen, 2007). They were discovered in Lee et al. (1993) and Wightman et al. (1993) in studies about the development of *Caenorhabditis elegans*. Gene expression control exerted by miRNAs is post-transcriptional as miRNAs regulate mRNA translation and stability in the cytoplasm (Valencia-Sanchez et al., 2006; Nilsen, 2007; Pillai et al., 2007). Human genome encodes more than 1000 miRNAs predicted to regulate over 60% of our genes (Friedman et al., 2009; Kozomara and Griffiths-Jones, 2014). Hence, miRNAs seem to participate in virtually every cellular process and changes in their expression are present in several human pathologies (Esquela-Kerscher and Slack, 2006; Krützfeldt and Stoffel, 2006; Chang and Mendell, 2007; Farazi et al., 2011).

Human adenoviruses were initially isolated from patients with acute respiratory infection but are now associated with many other pathological conditions such as gastroenteritis, keratoconjunctivitis, myocarditis, meningoencephalitis, cystitis, and hepatitis (Lenaerts et al., 2008). Despite its benign and self-limited course in imunocompetent host, adenoviral infections are particular severe in immunocompromised patient where a high morbidity and mortality could be observed (Lion, 2014). Adenoviruses have also been described as potential oncogenic viruses in rodents due do the presence of well-characterized oncogenes in viral genome, although its role in human carcinogenesis still basically unproven. Finally, human adenoviruses have recently been described as being able to establish life-long latent infections particularly in tonsilar and intestinal lymphocytes as well as in lung epithelial cells (Hogg, 2001; Garnett et al., 2009; Roy et al., 2011).

As described for many other viruses, adenoviral infection has a tremendous impact in cellular miRNA. Upon infection, host-cells miRNAs’ expression is severely altered together with viral blockage of key proteins acting in the silencing machinery and miRNA expression by adenoviruses (Carnero et al., 2011).

This review discusses the mechanisms underlying the biogenesis and molecular interactions of miRNAs providing basic concepts of their functions focusing in the interplay between miRNAs and adenoviruses.

## MicroRNA Biogenesis

The canonical miRNA biogenesis starts in the nucleus with the transcription of miRNA genes by RNA polymerase II or in some cases RNA polymerase III (Lee et al., 2004). These transcripts – primary-miRNA (pri-miRNAs) – consist of 1–6 hairpin structures containing a stem of about 33-nucleotides, a terminal loop and flanking single stranded RNA sequences (Cai et al., 2004). They contain a 5′-end cap and a poly-A tail sequence and are processed to functional miRNAs in two steps catalyzed by Drosha and Dicer. Both enzymes belongs to the RNase III family and function in complexes with dsRNA-binding proteins (dsRBPs).

The first step takes place in the nucleus and is catalyzed by Drosha and DiGeorge syndrome critical region gene-8 (DGCR8) (Lee et al., 2003). Pri-miRNAs are cropped by the Drosha-DGCR8 complex, also known as Microprocessor Complex, to precursor miRNAs (pre-miRNAs), 70-nucleotide long hairpin structures (Yan et al., 2003; Denli et al., 2004; Gregory et al., 2004; Han, 2004; Landthaler et al., 2004; Zeng et al., 2005). The DGCR8, a dsRBP, specifically recognizes and binds pri-miRNAs, acting as a ruler for Drosha to cleave pre-miRNAs in specific sites to hinder pre-miRNAs (Han et al., 2006). Some miRNAs can bypass processing by Drosha-DGCR8 complex. These are known as mitrons, spliced introns that correspond exactly to pre-miRNAs, both in length and structure, thus not needing the excision step by the Microprocessor Complex (Berezikov et al., 2007; Okamura et al., 2007; Ruby et al., 2007).

The second step of canonical miRNA processing occurs in the cytoplasm, hence the need of nuclear exporting of pre-miRNAs. A RanGTP-dependent dsRNA binding protein known as Exportin-5 binds pre-miRNA in the nucleus and releases it in the cytoplasm upon GTP hydrolysis to GDP (Yi et al., 2003; Bohnsack et al., 2004; Lund et al., 2004). Once in the cytoplasm, pre-miRNAs are processed by Dicer (Bernstein et al., 2001; Hutvágner et al., 2001). This final processing step leads to a 21- to 25- mature dsRNA, known as miRNA::miRNA^∗^ complex, that is ready for being loaded into a RNA-induced silencing complex (RISC) (Zamore et al., 2000). At this point, the RISC loading complex (RLC) forms and miRNAs are assembled into miRNA-induced silencing complex (miRISC). Beside Dicer, miRISC contains the Argonaute proteins (Ago) and two dsRBPs: TRBP (TAR RNA-binding protein) and PACT (protein activator of PKR), that are implicated in miRNA functions together with pre-miRNA processing (Chendrimada et al., 2005; Gregory et al., 2005; Haase et al., 2005; Lewis Phillips et al., 2008; Lee et al., 2013).

### miRNAs Target mRNAs Through Base Pairing

The binding between miRNAs and mRNAs is established through base-pair complementarity. In order to act as post-transcriptional regulators of gene expression, miRNAs must bind to their target(s) mRNA(s) through base-pair complementarity. In general, miRNAs pair imperfectly with target mRNAs in the 3′ untranslated region (3′ UTR). In this case, miRNAs must be perfectly and contiguously complementary in their nucleotides 2–8 at the 5′ end – the so called ‘seed’ region – in order to effectively supress their targets mRNAs (Doench, 2004; Brennecke et al., 2005). However, although important, the ‘seed’ region is not the only determinant for post-transcriptional repression of targeted mRNAs (Lewis et al., 2005; Grimson et al., 2007). In fact, we may define two main categories of miRNAs target sites: in the first category we include those that pair well in both 5′ and 3′ ends and those that require little or no additional 3′ pairing. In the second category, targets have weak 5′ base-pairing that is compensated by a strong pairing to the 3′ end (Brennecke et al., 2005). Apparently, additional 3′ pairing is required to induce miRNA-mediated target repression if complementarity with target mRNA is only based in the 5′ seed region.

Given the short length of the ‘seed’ region of miRNAs, it is obvious that one miRNA can target a large number of different mRNAs. This have been shown by microarray-analysis where the same miRNA can downregulate multiple mRNAs sharing one or more complementary sequences to the ‘seed’ region in their 3′ UTR (Lim et al., 2005). On the other hand, it is also been proved that more than one copy of the same miRNA or even several different miRNAs can pair with a single 3′ UTR from the target mRNA acting in a synergistic way. This synergic effect was demonstrated by adding multiple binding sites into a 3′ UTR: the result inhibition of translation was more efficient that expected from the sum of individual inhibition of each binding site (Doench, 2003). The mechanism of this synergism could be related with the mutual stabilization of different ribonucleoprotein complexes and/or with a more effective inhibition of translation (Doench, 2003). An obvious outcome of this cooperative interaction is that by regulating the degree of miRNA binding to the 3′ UTR of the mRNA will allow a cell to fine-tune mRNA expression.

### miRNAs Mediate Gene Repression by Avoiding mRNA Translation and Through mRNA Decay

The mechanisms by which miRNAs modulates mRNA expression are not yet fully understood. Despite the absence of conclusive answers, some aspects of these mechanisms are consensual.

MicroRNAs can modulate mRNA expression in two distinct ways: (i) by inhibiting translation or (ii) by destabilizing mRNAs. Recent studies indicate that these two mechanisms may occur sequentially (Fabian et al., 2009; Guo et al., 2010; Bazzini et al., 2012; Béthune et al., 2012; Djuranovic et al., 2012; Larsson and Nadon, 2013), being the destabilization the last and apparently the predominant step in miRNA-mediated mRNA repression. There are several proposed mechanisms for miRNA-mediated inhibition of translation, ranging from initiation and elongation interference, through ribosomal drop-off. From these, inhibition of translation initiation seems to be the predominant mechanism.

## Human miRNAs and their Functions

Considering that miRNAs can regulate up to 60% of human genes it is predictable that miRNAs are able to interfere with virtually all cellular pathways (Friedman et al., 2009). Given that a single miRNA can target 100s of genes (Lim et al., 2005), prediction of the functions of a single miRNA are made by identification of the most targeted pathways. Early reports have shown that miRNAs were able to target genes related with important cellular functions such as binding to nucleic acid, signal transduction and transcription regulation (Lewis et al., 2003). Not surprisingly, miRNA degradation and deregulation has been associated to many human pathologies such as pulmonary diseases (e.g., asthma, allergy, lung cancer) (Tomankova et al., 2010) and liver diseases (e.g., viral hepatitis, hepatocellular carcinoma) (Chen, 2009).

The deregulation of normal miRNAs expression or repression can influence several biologic processes, including carcinogenesis. One good example is hsa-miR-155, a miRNA associated with oncogenic processes when upregulated. However, hsa-miR-155 is important for proper immune system functioning as it regulates immune cells activation, growth and inflammatory cytokines release (Mashima, 2015). It was demonstrated that hsa-miR-155 has critical roles in both innate and adaptive immune responses, as well as in the balance between immune response and immune tolerance. Several studies show that hsa-miR-155 regulates (i) the differentiation of T-CD4+ lymphocytes into different subsets of helper T-cells (i.e., Th1, Th2, and Th17), (ii) the development of regulatory T-cells, (iii) the activation of T-CD8+ lymphocytes, and (iv) the differentiation of B lymphocytes and antibody production (Seddiki et al., 2014). Besides hsa-miR-155, many other miRNAs have been associated with T-cell activation, differentiation and expansion, namely hsa-miR-181a, hsa-miR-146a, hsa-miR-182, hsa-miR-17-92, and hsa-miR-125 (Baumjohann and Ansel, 2013).

Bacterial infections have a tremendous impact in cellular miRNA. For example, upon *Mycobacterium tuberculosis* (Mtb) infection, host-cells miRNAs’ expression is severely altered with potential implications in survival and pathogenesis of *Mycobacterium tuberculosis* (Bettencourt et al., 2015). *Helicobacter pylori* was also referred has being able to interfere with hsa-miRNA-155 expression in gastric epithelial cells (Xiao et al., 2009).

Interestingly, miRNAs can have different and somehow opposite functions depending on the cellular context. The hsa-miR-125 is one of these miRNAs. In fact, it can act as an oncogenic or as tumor suppressive miRNA in prostate and breast cancer, respectively (Sun et al., 2013). Hsa-miR-125 is known to target diverse pathways such as differentiation, apoptosis, immune response, cell proliferation, and metastasis in carcinogenic processes (Sun et al., 2013). Lastly, some miRNA clusters cooperate for the same function. It is the case of hsa-miR17/92 cluster, also known as oncomir1, which is also involved in oncogenic processes. This miRNA cluster encodes six miRNAs that cooperatively target PTEN (phosphatase and tensin homolog), SMAD, TGFBR2 (transforming growth factor, beta-receptor II) and P21 genes, which are related to cell growth and proliferative signaling (Fuziwara and Kimura, 2015).

## Role of Cellular miRNAs in Viral Infections

Interactions between virus and host are intricate and complex. Due to their intracellular replication cycle it is no surprise that virus evolved in order to create mechanisms allowing them to use or avoid host-encoded miRNAs to infect, survive and replicate in host cells (Grundhoff and Sullivan, 2011; Harwig et al., 2014).

Mutual interference mechanisms between viruses and host-cell’s miRNA machinery have been described. To generate a more favorable cellular environment or to regulate their own miRNAs, viruses can (i) avoid cellular miRNAs targeting viral mRNAs (Cullen, 2013), (ii) block or impair the miRNA pathway by interacting with some key proteins (Lu and Cullen, 2004; Bennasser et al., 2006), (iii) synthesize their own miRNA (Grundhoff and Sullivan, 2011; Harwig et al., 2014), (iv) or make use of cellular miRNAs to their favor (Luna et al., 2015). Conversely, host-cell’s endogenous miRNAs are also able to target viral mRNAs (Lecellier et al., 2005; Delorme-Axford et al., 2013; Bai and Nicot, 2015).

In general, cellular miRNAs repress viral gene expression resulting in decreased viral replication; this viral repression can contribute to viral latency. However, cellular miRNAs can also increase viral replication and some viruses, such as Hepatitis C Virus (HCV), seem to depend on cellular miRNAs to replicate efficiently. In fact, although some cellular miRNAs (e.g., hsa-miR-181c, hsa-miR-196, hsa-miR-199a, hsa-miR-488, and hsa-miR-let-7b) interact directly with viral genome inhibiting HCV replication (Piedade and Azevedo-Pereira, 2016b), one liver-specific miRNA, hsa-miR-122, binds to 5′ UTR region of HCV RNA enhancing viral genome replication and accumulation in infected cells (Jopling et al., 2005).

Several host-cells’ miRNAs have been described with the capacity to block different viral replication steps. Although some of these data are still controversial, there is a growing body of evidence pointing to an antiviral mechanism mediated by miRNA. These mechanisms could involve a direct interaction with a viral protein or an indirect interaction with some cell protein required to a certain viral replication step. Several examples can be given within viral infections:

(i) In HIV-1 infection, several host miRNAs have been described able to interact with HIV host dependency factors (HDFs). These HDFs are key players in HIV-1 cycle and their repression generally results in repressed viral replication (reviewed in Piedade and Azevedo-Pereira, 2016b). The activation of TLR3 and TLR4 during HIV replication cycle upregulates hsa-miR-155 that targets and repress ADAM10 (disintegrin and metalloproteinase domain-containing protein 10), Nup153 (nucleoporin Mr 153,000), TNPO3 (transportin 3) and LEDGF/p75 (lens epithelium-derived growth factor), all of them important HDFs involved in nuclear import and integration of HIV genome (Swaminathan et al., 2012; Seddiki et al., 2014). Similarly, VprBP (Vpr binding protein), a cellular factor crucial for Vpr-mediated G2 cell-cycle arrest and proper viral replication, is targeted by hsa-miR-1236 (Ma et al., 2014).
Additionally, host cell miRNAs were described targeting HDFs important for HIV-1 Tat-mediated LTR activation: PCAF (P300/CBP-associated factor), a histone acetylase is target by hsa-miR-20a and hsa-miR-17-5p (Triboulet et al., 2007); purine-rich element binding protein alpha (PUR-α), is targeted by hsa-miR-15a, hsa-miR-15b, hsa-miR-16, hsa-miR-20a, hsa-miR-93, and hsa-miR-106b (Shen et al., 2012); and cyclin T1 is repressed by hsa- miR-198 and hsa-miR-27b (Sung and Rice, 2009; Chiang et al., 2011).
(ii) Placental trophoblasts are highly resistant to infection by several and unrelated viruses, including human cytomegalovirus (HCMV) herpes simplex virus-1, poliovirus, coxsackievirus B3, vesicular stomatitis virus, and vaccinia virus (Delorme-Axford et al., 2013). This resistance is conferred by a group of miRNAs from a cluster located in chromosome 19. Apparently these miRNAs are expressed in human placental trophoblasts and transferred to non-placental cells by an exosome-mediated mechanism, rendering recipient cells resistant to viral infection.
(iii) Cellular miRNAs can also interfere with viral infection by regulating innate response after TLR sensing of virus-associated nucleic acid. Hsa-miR-126 was found to regulate the expression of TLR7 and TLR9 in plasmacytoid dendritic cells (pDC), in addition to other molecules involved in signaling pathways crucial for type I IFN-mediated innate immune response (Agudo et al., 2013). This regulation of pDC response could be of paramount importance during HIV interaction with dendritic cells after sexual mucosal transmission (Barroca et al., 2014).
(iv) In herpesviruses infections several cell-encoded miRNAs control latent/lytic cycles. For example, hsa-miR-200b and hsa-miR-429 regulate Epstein–Barr virus (EBV) and HCMV switch from latent to lytic infection (reviewed in Piedade and Azevedo-Pereira, 2016a).
(v) Another example of cell-encoded miRNAs that may promote viral persistence and immune escape by silencing the expression of viral genome is hsa-miR-28-3p. The target sequence of this cellular miRNA is located within the *gag/pol* gene of human T-lymphotropic virus-1 (HTLV-1) and reduces viral replication and gene expression (Bai and Nicot, 2015). Interestingly, hsa-miR-28 is upregulated by IFN response revealing a mechanism where innate immune response helps viral persistence by reducing virus dissemination to neighboring cells, diminishing local inflammation, and enabling the survival of infected cells.
(vi) Finally, cellular miRNAs were predicted to target several HIV-1 transcripts, thus reducing viral replication (Piedade and Azevedo-Pereira, 2016b). However, these predictions not always translate in effective repression due the multiplicity of factors that are involved in miRNA-mediated gene regulation. For example, HIV-1 encodes RNA interference silencing suppressors that interfere with miRNA pathways: HIV-1 Tat suppresses Dicer function, and Nef viral protein decreases miRNA function by directly binding to Ago2. Noteworthy, Nef stimulates the secretion of several host-cell’s miRNAs in Nef-containing exosomes. Additionally, HIV-1 is able to evade miRNAs by two important mechanisms: the secondary structure of HIV transcripts (Westerhout et al., 2005; Watts et al., 2009), and the outstanding genetic variability both in length and sequence (Bandaranayake et al., 2010). Both strategies render HIV-1 RNAs resistant to host miRNAs.

## Role of Virus-Encoded miRNAs in Pathogenesis of Viral Infections

Some viruses encode miRNAs that have an important role in infection and pathology. The functions of these viral miRNAs vary with different viruses and in different tissues but some pathways are often targeted, suggesting that virus evolved separately toward similar purposes. Although the overall influence of miRNAs in the pathogenesis of viral infection is beyond the scope of this review, it is important to highlight that viral miRNAs were reported to regulate genes related with immune response, apoptosis, cell cycle control, differentiation, and intracellular trafficking. As many of these altered pathways are of the uttermost importance for cellular homeostasis, their alterations largely contribute for viral pathology.

One of the most noteworthy pathological events triggered by viral miRNAs is oncogenesis. In fact, oncogenic viruses such as EBV and Kaposi’s sarcoma-associated herpesvirus (KSHV) encode viral miRNAs that can be directly linked with development of malignancies (reviewed in Zhu et al., 2013; Piedade and Azevedo-Pereira, 2016a). For example, different EBV-encoded miRNAs (BART3-5p, BART7, and BART19-3p) target several tumor-suppressors genes facilitating typical B-cell transformation (Wong et al., 2012; Lei et al., 2013), and several KSHV miRNAs cooperatively regulate cytokine expression, cell survival, and cellular transcription factors to facilitate infection and oncogenesis while avoiding the host immune system (Piedade and Azevedo-Pereira, 2016a).

Viral miRNAs can also target viral transcripts, particularly in herpesviruses infections. Repression of viral genes is an efficient strategy to maintain latency and to keep viral loads to minimum, avoiding detection by host defenses. Viral miRNAs can also be considered important switches between lytic and latent infection and their levels in the host cell help to determine the evolution of the viral infection.

Another mentionable feature of viral miRNAs is that they seem to have evolved to target conserved regions of the host genes and even specific sequences targeted by host miRNAs. These strategies avoid potential mutations in the recognition sequences of target mRNAs, ensuring viral miRNA function. Thus, these evolutionary paths are suggestive of the importance of viral miRNAs for establishment and prevalence of viral infection.

## Human Adenovirus Encode Virus-Associated RNA with Pro-Viral Functions

**FIGURE 1:**
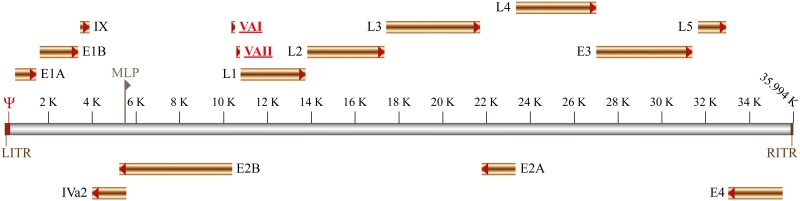
Schematic representation of adenovirus genome organization. The early and late genes are shown as well as the left and right Inverted Terminal Repeat (RITR and LITR, respectively). Both viral DNA strands (represented in gray) are transcribed: in the top are indicated the genes transcribed from the ‘rightward’ reading strand (E1A, E1B, IX, L1-L5, and E3), whereas the genes transcribed from the ‘leftward’ reading strand are shown bellow (E4, E2A, E2B, and IVa2). The packaging signal (Ψ) and the major late promoter (MLP) are also indicated. The viral-associated non-coding RNAs I and II (VAI and VAII respectively) are represented in red and underlined.

**FIGURE 2:**
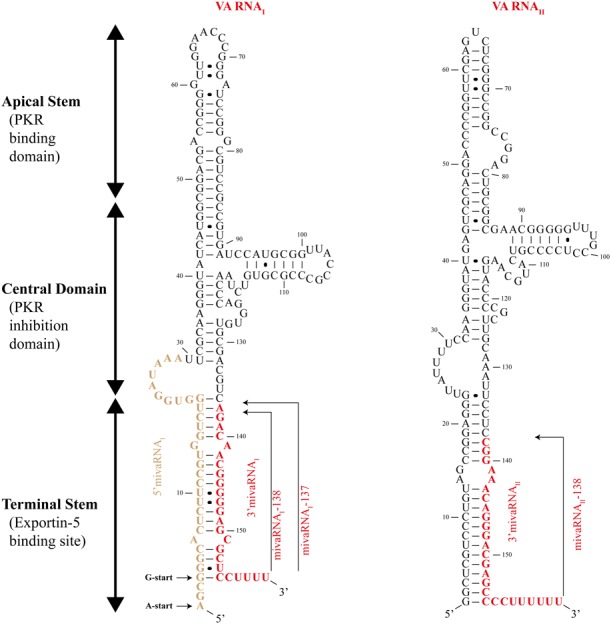
Structure of adenovirus virus-associated RNAs. Schematic drawing showing the structure of adenovirus virus-associated (VA) RNA_I_ **(left)** and VA RNA_II_ **(right)**. The three structural domains – apical stem, central domain, and terminal stem – are indicated. The viral miRNAs (mivaRNA_I_-138, mivaRNA_I_-137, and mivaRNA_II_-138) are represented in red. The VA RNA_I_ could start from two different sites indicated by the A-start and G-start arrows. Black circles between nucleotides represent non-Watson-Crick base pairing.

Human adenoviruses genome encodes two non-coding RNAs, transcribed by cellular RNA polymerase III known as viral-associated (VA) RNAs (**Figure 1**). These two VA RNAs have similar lengths, VA RNA_I_ is 157–160 nucleotides long and is found in every adenovirus while VA RNA_II_ is 158–163 nucleotides long and is found in 80% of adenoviruses, including the serotype 5 (Vachon and Conn, 2015). Although the primary nucleotide sequence of VA RNAs may differ between adenoviruses, they show a highly conserved secondary structure (Ma and Mathews, 1996). The secondary structure can be divided in three structural domains (**Figure 2**): (i) Terminal Stem, (ii) Central Domain, and (iii) Apical Stem (Coventry and Conn, 2008). These different structures are associated with distinct functions of VA RNAs as they interact and bind different cellular factors: the Apical Stem binds to PKR; the Central Domain inhibit PKR; and the Terminal Stem binds to Exportin-5 (**Figure 2**). These functions are essential for proper viral replication. Deletion of both VA RNAs caused a 60-fold decrease in viral replication rate while deletion of VA RNA_I_ showed a reduction of 10–20 fold (Thimmappaya et al., 1982; Bhat and Thimmappaya, 1984). Interestingly, deletion of VA RNA_II_ did not cause a measurable reduction of viral viability (Thimmappaya et al., 1982) suggesting that VA RNA_II_ absence can be partially compensated by VA RNA_I_ and might exert non-essential functions while VA RNA_I_ is the predominant pro-viral VA RNA (Carnero et al., 2011).

The first identified function of VA RNA_I_ (**Table 1**) was the inhibition of innate immune response mediated by the protein double-stranded RNA-activated kinase (PKR) (Kitajewski et al., 1986). This interferon-induced kinase is activated by dsRNA (including viral RNA) and phosphorylates the α subunit of the eukaryotic initiation factor 2 alpha (eIF2α) which in turn prevents the eIF2β of being recharged with GTP, avoiding the formation of the 43S pre-initiation complex following translation initiation (Krishnamoorthy et al., 2001; Cole, 2007). VA RNA_I_ avoids translational blockage by inactivating PKR, allowing the production of viral proteins. The inactivation of PKR is mediated by the central domain of VA RNA_I_, while the apical domain is required for efficient PKR binding (Launer-Felty and Cole, 2014).

**Table 1:** Adenovirus non-coding RNAs.

Adenovirus non-coding RNAs	Cellular targets	Predicted effects	Reference
VA RNA_I_	PKR	Inhibition of innate immune response	Kitajewski et al., 1986; Launer-Felty and Cole, 2014
	RIG-I	Activation of type I interferon	Minamitani et al., 2011
	OAS1	Protein synthesis arrest	Desai et al., 1995
VA RNA_I_ and VA RNA_II_^∗^	Exportin-5	Interference with the miRNA pathway by competition with pre-miRNAs for Exportin-5	Lu and Cullen, 2004; Bennasser et al., 2011
	Dicer	Interference with the miRNA pathway by decreasing cellular levels of Dicer and saturation of Dicer	Lu and Cullen, 2004; Bennasser et al., 2011
	RISC	Target the miRNA pathway by interfering with RISC’s assembly and function	Andersson et al., 2005; Xu et al., 2007, 2009
mivaRNAI	Ly6K	Deregulation of cell growth	Aparicio et al., 2010; Bellutti et al., 2015
	TIA1	Regulation of apoptosis; potential switch between early and late stages of adenoviral infection	Aparicio et al., 2010; Carnero et al., 2011

*Summary of cellular targets and predicted effects of VA RNAs and mivaRNAs encoded by adenovirus. ^∗^Given the described saturation of Dicer and competition for Exportin-5, it would be expected that adenoviral VA RNAs lead to a general downregulation of cellular miRNAs, and thus to an increase in their targets.*

Besides PKR, VA RNA_I_ also interacts with other innate immune system factors (**Table 1**) such as retinoic acid-inducible gene 1 (RIG-I) (Minamitani et al., 2011) and these interactions seem to activate type I interferon. This may seem contradictory with PKR inhibition by VA RNA_I_ and is hypothesized that other viral factors are able to inhibit RIG-I (Punga et al., 2013). Similarly, the 2′-5′ oligoadenylate synthase-1 (OAS1), another dsRNA-sensor, is also activated by VA RNA_I_ (Desai et al., 1995). OAS1 activates RNase L pathway that leads to viral protein synthesis arrest through the degradation of viral RNA (**Table 1**). Again, this pathway would enhance virus restriction in conflict with PKR inhibition. Some data may help solve this contradiction: first, VA RNA_I_ binding and activation of OAS1 is very inefficient when compared with a dsRNA of the same size (Desai et al., 1995). Furthermore, truncated VA RNA_I_ containing apical stem and central domain – thought to be the result of Dicer cleavage, as will be described – shows an increased affinity for OAS1. These observations might indicate that in the course of adenoviral infection, the truncated form of VA RNA_I_, acting as a pseudo-inhibitor, will outcompete with full-length VA RNA_I_ leading to a block of OAS1 activation, thus inhibiting several innate immune response pathways (Meng et al., 2012).

## VA RNAs Can Overload Exportin-5 and Dicer Disrupting Cellular miRNA Functions

Despite being well-known for its pro-viral activity, VA RNAs can also play an important role in the deregulation of host miRNAs (**Table 1**). VA RNAs interfere with miRNA pathway on three different levels: (i) competition with pre-miRNAs for Exportin-5, (ii) saturation of Dicer, and (iii) interference with RISC’s assembly and function.

Upon transcription by RNA polymerase III, VA RNA_I_ is exported from the nucleus to the cytoplasm by Exportin-5. This RanGTP-dependent dsRNA binding protein recognizes VA RNA_I_ secondary structure (more precisely, the Terminal Stem; **Figure 2**) which is similar to pre-miRNAs (Gwizdek et al., 2003). The VA RNA_I_-Exportin-5 binding is highly efficient and Exportin-5 is thought to be quickly saturated by the VA RNA_I_ leading to the blockage of pre-miRNAs export from the nucleus (Lu and Cullen, 2004). Furthermore, Exportin-5 is also responsible for export Dicer mRNA into the cytoplasm, thus VA RNA_I_ saturation of Exportin-5 can further interfere with miRNA pathway through the decrease of cellular levels of Dicer (Bennasser et al., 2011).

Once in the cytoplasm, VA RNAs bind and sequester Dicer preventing it from maturing cellular pre-miRNAs (Lu and Cullen, 2004). VA RNAs are also processed by Dicer and originate two distinct fragments: Apical Stem-Central Domain (AS-CD), and Terminal Stem which originates viral miRNAs, known as mivaRNAs (**Figure 2**), that will be loaded into RISC (Andersson et al., 2005; Aparicio et al., 2006; Sano et al., 2006; Xu et al., 2007). AS-CD fragments could not be detected probably due to their instability after Dicer processing (Aparicio et al., 2006). Interestingly, VA RNA is inefficiently processed by Dicer as only 2–5% of total VA RNA_I_ is cleaved (Aparicio et al., 2006). However, there are approximately 10^8^ molecules of VA RNA_I_ per cell, meaning that over 10^6^ molecules of mivaRNAs are perfectly functional and able to act as miRNAs when loaded into RISC (Andersson et al., 2005; Aparicio et al., 2006). Despite having 20-fold less expression than VA RNA_I_, VA RNA_II_ seems to be the preferential substrate for Dicer processing originating twice the mivaRNAs compared to VA RNA_I_ (Xu et al., 2007).

The unprocessed VA RNA_I_ can give raise to distinct mivaRNAs depending on the cleavage site of Dicer, being mivaRNA_I_-137 and mivaRNA_I_-138 (named after their starting nucleotide in the VA RNA_I_ molecule) the two most abundantly produced (Xu et al., 2007). Furthermore, the transcription of VA RNA_I_ could start from two different sites giving rise to VA RNA_I_(G) and VA RNA_I_(A) – according to their first nucleotide (**Figure 2**) – and thus to even more mivaRNAs (Xu et al., 2009).

It was demonstrated that after being processed by Dicer, the 22 nucleotide-long mivaRNAs derived from VA RNAs can incorporate RISC (Lu and Cullen, 2004; Andersson et al., 2005; Aparicio et al., 2006; Sano et al., 2006). Furthermore, mivaRNA_II_, derived from VA RNA_II_, was found associated with polyribosomes, indicating the ability to regulate gene expression (Xu et al., 2007). Another compelling evidence pointing to mivaRNAs as functional miRNAs, was the finding that 80% of RISC complexes contain mivaRNAs in late adenoviral infections (Xu et al., 2007), though a more recent report points to a smaller proportion of RISC occupied by mivaRNAs (Bellutti et al., 2015). Despite these somehow contradictory evidences, the sheer numbers of mivaRNAs and their ability to be incorporated into RISC is thought to be enough to interfere with cellular mRNA. As mentioned above, mivaRNA_II_ seems to be the major mivaRNA to be associated with RISC (Andersson et al., 2005; Aparicio et al., 2006), yet the seed sequences of mivaRNA-containing RISC did not match the ones from mivaRNA_II_ (Bellutti et al., 2015) and mivaRNA_I_ was shown to reduce more efficiently the expression of a reporter gene included in constructs containing the mivaRNA_I_ target regions (Bellutti et al., 2015). These findings are consistent with reports of asymmetrical loading of mivaRNAs into RISC. In fact, 5′- and 3′-end strands of mivaRNA_I_(A) and mivaRNA_I_(G) are not equally incorporated into RISC as the 5′ strand of mivaRNA_I_(G) is not found in RISC (Andersson et al., 2005; Aparicio et al., 2006).

Potential mivaRNA targets were evaluated through different approaches (Aparicio et al., 2010; Bellutti et al., 2015). Based on the results obtained, several genes with essential cellular functions were deemed direct or indirect targets of mivaRNAs (**Table 1**). The cellular functions apparently affected by mivaRNAs are cell signaling (seven genes), cell growth and apoptosis (nine genes), DNA transcription or repair (nine genes) and RNA metabolism (five genes) (Aparicio et al., 2010). One confirmed target of mivaRNA is the protein Ly6K (lymphocyte antigen 6 complex, Locus K) (Bellutti et al., 2015), a protein associated with cell growth and implicated in several cancers such as lung, breast or head and neck (Vachon and Conn, 2015). Another target of mivaRNAs is T-Cell-Restricted Intracellular Antigen-1 (TIA1), a RNA-binding protein, regulator of proapoptotic molecules (Aparicio et al., 2010). This protein is associated with stress granules and binds to U-rich sequences in mRNAs, abundant in adenoviral transcripts, increasing splicing or blocking translation (Carnero et al., 2011). In fact, it is hypothesized that downregulation of this protein may be related with alternative splicing of adenoviral transcripts, such as E1A, being a potential switch between early and late stages of infection (Carnero et al., 2011). Together, these two genes provide an example of pro-viral function of mivaRNAs in infected cells. However, whether the genes targeted by mivaRNAs are essential for viral infection or not is still uncertain (Kamel et al., 2013; Vachon and Conn, 2015). Besides targeting cellular mRNAs, it was expected that mivaRNAs could also target viral transcripts in order to regulate viral life cycle, similar to other viruses, such as herpesviruses (Piedade and Azevedo-Pereira, 2016a). Remarkably, this seem not be the case with adenoviruses since bioinformatics scanning of viral genome for potential mivaRNA targets found no matches (Aparicio et al., 2006).

Adenoviruses code for small RNAs other than VA RNAs and respective mivaRNAs. Interestingly, some of these small RNAs overlap mivaRNA sequences and are synthesized prior to VA RNAs expression. The different patterns of expression between viral small RNAs and mivaRNAs probably signifies different functions as none of the small RNAs detected had the canonical size of miRNAs (Zhao et al., 2013).

## Adenovirus Deregulate Cellular miRNAs in Order to Favor Viral Replication

Viral interference with miRNAs goes beyond mivaRNAs effect on putative target expression. In fact, adenoviruses are able to deregulate cellular miRNA levels (Qi et al., 2010; Su et al., 2010; Zhao et al., 2015). Given the large amount of VA RNAs expressed and the subsequent blockade of Dicer and Exportin-5, it would be expected that adenoviral infection lead to a general downregulation of cellular miRNAs, and thus to an increase in their targets. However, this does not occur during the first hours of adenovirus infection since VA RNA accumulation is not immediate and other factors, both cellular and viral, contribute to differential expression of miRNAs (Qi et al., 2010; Zhao et al., 2015). The differential expression of cellular genes appears to follow four different stages upon infection by adenoviruses. The first stage goes from 0 to 12 h post-infection, when the viral gene expression starts and cell growth is inhibited as an host defense against the virus (Granberg et al., 2006; Zhao et al., 2007). The second stage goes from 12 to 24 h post-infection and is marked by expression of E1A viral gene, which contributes largely for changes in cell’s environment that contribute to optimal viral replication. Most of downregulated genes at this stage are related to cell cycle regulation, cell proliferation, and antiviral response. The third stage follows until 36 h post-infection when replication of the viral genome takes place upon full control of cellular metabolism. The fourth and last period is characterized by a general and marked deregulation of cellular genes. At this stage, more than 3700 genes were identified as being deregulated by at least twofold their normal expression (Zhao et al., 2012).

Not surprisingly, deregulation of miRNA also appears to follow this stepped progression, with miRNA expression varying from up to downregulation and vice versa (Qi et al., 2010; Zhao et al., 2015). A recent study reported changes in 175 miRNAs expression above 1.5-fold normal expression (Zhao et al., 2015). This study showed that most deregulated miRNAs during early infection (<24 h post-infection) were upregulated. From those, the most highly expressed were known to target either tumor suppressor or immune response genes. Namely, hsa-miR-22, a cell growth inhibitor, hsa-miR-181b, hsa-miR-320 and hsa-let-7e, all tumor suppressor miRNAs, were all upregulated in the first 6 h post-infection as part of the host-cell immune response to the virus. Another interesting miRNA upregulated during the first 6 h post-infection was hsa-miR-155, a well-studied oncomir, that is hypothesized to play a role in host antiviral response (Zhao et al., 2015). After 12 h of infection a second wave of cellular antiviral miRNAs are expressed. Among these overexpressed miRNAs are hsa-miR-29 and hsa-let-7d, both involved in immune response. Simultaneously, oncogenic miRNAs, such as hsa-miR-21 and miRNAs from hsa-miR-17/92 cluster (oncomir-1) are downregulated. Also at this point, the first miRNAs to be regulated by the virus, opposing cellular efforts against infection, is detected. Oncogenic miRNA hsa-miR-574 is upregulated and tumor suppressor miRNAs such as hsa-let-7i, hsa-miR-34a, hsa-miR-185 and hsa-miR-31 are downregulated (Zhao et al., 2015). The involvement of E1A, VA RNAs and respective mivaRNAs are the probable cause for this, apparently contradictory, pattern of expression.

During the third period of viral infection, deregulation of cellular miRNAs reaches a turning point when viral transcripts completely overcome host defenses. At this stage, 24 h post-infection, the switch between early and late infection occurs with an apparent control of cellular pathways by viral proteins and VA RNAs. The cell-encoded miRNA expression profile changes and almost all cellular miRNAs that were previously overexpressed become downregulated. Among them are hsa-miR-22, hsa-miR-181b and hsa-miR-320 that were overexpressed at 6 and 12 h post-infection as part of the host immune response to the virus. Other cellular miRNAs associated with tumor suppressive and immune-modulating functions, such as hsa-miR-143, hsa-let7a/b and hsa-miR-29a, were downregulated at this point of infection after being highly expressed during early stages. Despite the general downregulation of miRNAs, some were found upregulated at 24 h post-infection. From these, the vast majority were transiently expressed being downregulated soon after, at 36 h post-infection, by late viral transcripts. Two cell-encoded miRNAs, hsa-miR-27a and hsa-miR-125b (as well as hsa-miR-27b and hsa-miR-125a, although in less extent), are particularly important to refer in this context given their role as oncogenic or tumor suppressive miRNAs, depending on the tissue type, and their interference with viral replication during infection by other viruses such as HCV, HCMV, and HPV. These cellular miRNAs are upregulated at 24 h post-infection and are suppressed during late stages of adenoviral infection as mentioned above. Together with hsa-miR-199a and hsa-miR-140, known for their impairment of tumor growth, they may stand as host cell last line of antiviral defense. Interestingly some oncomirs were found downregulated at this time point, something not expected given the miRNA profile. Whether the suppression of these miRNAs, such as hsa-miR-193 and hsa-miR-221, is a consequence of viral activity or stands as yet another cellular defense against the virus remains to be clarified (Zhao et al., 2015).

After 36 h of infection, only five miRNAs were upregulated, all of them known as being oncogenic. From the remaining deregulated miRNAs, the most significant were hsa-miR-23/27 cluster and hsa-let-7, hsa-miR-30, and hsa-miR-376 families. These combination of miRNAs have established or predicted properties as tumor suppressors, cell proliferation inhibitors or apoptosis inducers (Zhao et al., 2015). Interestingly, a previous study found only 80 differentially expressed miRNAs after 72 h of infection (Qi et al., 2010). The most noteworthy miRNAs referred in both studies were hsa-miR-27a/b, hsa-miR-30a/b/c, hsa-miR-125a/b, hsa-miR-181b, and hsa-let-7e (Qi et al., 2010; Zhao et al., 2015). All of them have important roles as antiviral, tumor suppressive or oncogenic miRNAs. However, since there are several possible targets for these deregulated cellular miRNAs, and some are differentially expressed depending the tissue or the cell type (e.g., cancer cell vs. non-transformed cell), it is difficult to anticipate the outcome of their decreased or increased expression.

## Effect of Host Cell miRNAs on Adenoviral Infection

As for many other viruses, adenovirus replication could also be inhibited by cell-encoded miRNA. Considering the basic mechanisms of viral evolution, those cellular miRNA that are specifically deregulated during adenovirus infection should constitute those that have more pronounced effects on viral replication. Not surprisingly, hsa-miR-27 was recently described as a potent adenovirus inhibitor (Machitani et al., 2017). Adenovirus replication seems to be inhibited *via* the suppression of SNAP25 (synaptosomal-associated protein, 25 kDa) – associated with membrane fusion (Sudhof and Rothman, 2009) – and TXN2 (thioredoxin, mitochondrial, also known as thioredoxin-2) – a redox-active protein playing important roles in the regulation of mitochondrial redox and the production of reactive oxygen species with direct effects on redox balance, cell growth, and apoptosis (Tanaka, 2002; Conrad et al., 2004).

The post-transcriptional silencing of both genes by hsa-miR-27, lead to efficient suppression of adenovirus replication by two distinct mechanisms: silencing of SNAP25 interferes with adenovirus entry into target cells, while TXN2 suppression hampers adenovirus replication through a G1 arrest of cell cycle (Machitani et al., 2017). Due to the role of SNAP25 in endocytic intracellular trafficking and since adenovirus enter the cell through endocytosis (Meier and Greber, 2003) it is conceivable that suppression of SNAP25 expression will affect virus entry. Conversely, adenovirus replication relies on the induction of cell cycle transition from G0 or G1 to S phase in order to create optimal conditions for viral replication (Ben-Israel, 2002). Therefore, the arrest on G1 phase due to TXN2 suppression predictably imposes a non-productive adenoviral infection.

Conversely, cellular miRNAs can also induce adenovirus replication. Hsa-miR-26b has been identified as a potent stimulator of human adenovirus serotype 5 (Ad5) replication and propagation in prostate cancer cells (Hodzic et al., 2017). Apparently, an hsa-miR-26b-dependent NF-kB inhibition is one of the mechanisms underlying the enhancement of adenovirus replication. The identification of a cell miRNA promoting adenovirus replication in cancer cells is particularly important since adenoviruses have been used as tumor-lysing therapeutics (oncolytic virotherapy) as well as immune-stimulating vectors aiming to induce an immunogenic tumor cell death (Lichty et al., 2014; Rosewell Shaw and Suzuki, 2016). Although it is difficult to fully anticipate all the consequences of miRNA-mediated gene suppression, any mechanism that may potentiate the replication of these adenovirus-based vectors should be considered for the improvement of such therapies.

## Conclusion

Given the complexity of the interaction between host-cell and virus is difficult to realize all the players during viral infection. Adenoviruses evolved complex mechanisms to overcome host defenses. One of the most remarkable of these mechanisms is the ability to interfere with host miRNAs (Carnero et al., 2011; Vachon and Conn, 2015). VA RNAs and derived mivaRNAs seem to play an important role either by blocking important proteins from miRNA pathway, such as Exportin-5 and Dicer, or by targeting relevant cellular factors, such as TIA1 and cell-growth protein Ly6K (Vachon and Conn, 2015). Drastic changes in cellular miRNA expression profile are also noticeable and several cellular functions are affected by these changes as confirmed by transcriptome analysis and miRNA target prediction (Zhao et al., 2012, 2015). Most of the differentially expressed miRNAs are related to cell proliferation, apoptosis, cell signaling and immune response, contributing to a more favorable host environment for viral replication (Zhao et al., 2015). It will be interesting to understand if mivaRNAs and VA RNAs have an important role in this deregulation of cellular miRNAs or if the effect of viral miRNAs is restricted to gene suppression. In addition, it is important to clarify the importance of mivaRNAs in viral pathogenesis. The expression of miRNA and miRNA-mediated regulation of gene expression is relatively slow, being only noticeable after 24 h of infection (Qi et al., 2010; Zhao et al., 2015). Moreover, it is conceivable that miRNA regulation by adenoviruses could be more important for persistent rather than lytic infection, as it has been suggested by the association of VA RNAs with latent infection by adenoviruses (Xu et al., 2007). Finally, some cell miRNAs impact adenovirus replication cycle, either with pro-viral or antiviral activities. As the complex interactions between adenovirus and miRNAs are being uncovered, a better understanding should be achieved to fully elucidate the role of cell-encoded miRNAs in the regulation of adenovirus infection, particularly in the context of oncolytic therapy using adenovirus-based vectors.

## Author Contribution

All authors listed have made a substantial, direct and intellectual contribution to the work, and approved it for publication.

## Conflict of Interest Statement

The authors declare that the research was conducted in the absence of any commercial or financial relationships that could be construed as a potential conflict of interest.
